# Diagnostic Accuracy of Positron Emission Mammography with ^18^F-fluorodeoxyglucose in Breast Cancer Tumor of Less than 20 mm in Size 

**DOI:** 10.22038/AOJNMB.2018.31101.1213

**Published:** 2019

**Authors:** Fuzuki Yano, Masatoshi Itoh, Hisashi Hirakawa, Seiichi Yamamoto, Akira Yoshikawa, Jun Hatazawa

**Affiliations:** 1 Department of Nuclear Medicine and Tracer Kinetics, Osaka University, Graduate School of Medicine, Osaka, Japan; 2 Sendai Medical Imaging Clinic (Gazo Kenshin), Sendai, Japan; 3 Department of Breast Surgery, Tohoku Kosai Hospital, Sendai, Japan; 4 Radiological and Medical Laboratory Sciences, Nagoya University, Graduate School of Medicine, Nagoya, Japan; 5 Institute for Materials Research, Tohoku University, Sendai, Japan

**Keywords:** Breast cancer, Diagnostic accuracy, PEM, PET/CT, Positron emission mammography

## Abstract

**Objective(s):**

To investigate the diagnostic accuracy of positron emission mammography (PEM) and positron emission tomography/computed tomography (PET/CT) for small breast tumors of less than 20 mm in size.

**Methods:**

The study was conducted on a total of 100 subjects (i.e., 50 patients with pathologically proven breast cancer and 50 normal cases of medical screening). The total number of tumors was 54 (mean size: 11±5.1 mm, range: 4-20 mm). The diagnostic accuracy of PEM alone, PET/CT alone, and combined PET/CT and PEM was evaluated by two nuclear medicine physicians based on visual inspection. The two groups (i.e., tumors of ≤ 10 mm and > 10-20 mm) were compared in terms of the diagnostic capability of the three modalities (PEM alone, PET/CT alone, and PET/CT+PEM).

**Results:**

The sensitivities of PEM alone, PET/CT alone, and combined PET/CT and PEM were 72%, 60%, and 76%, respectively. The specificities of these tests were 98%, 100%, and 98%, respectively. Furthermore, the accuracies of these diagnostic modalities were 85%, 79%, and 87%, respectively. The combined PET/CT and PEM showed significantly higher sensitivity and accuracy than PET/CT alone (P=0.005 and P=0.02, respectively). In addition, PEM demonstrated a significantly higher sensitivity than PET/CT in the ≤ 10 mm group (P=0.03); however, no difference was observed between the two modalities in the > 10 mm group in terms of sensitivity.

**Conclusion:**

^18^F-fluorodeoxyglucose PET had limited capability for the detection of small breast cancers of < 10 mm. Combined PET/CT and PEM showed higher sensitivity and accuracy, compared to PET/CT alone.

## Introduction

^18^F-fluorodeoxyglucose (^18^F-FDG) positron emission tomography (PET) is a non-invasive diagnostic modality with a wide clinical application. This imaging technique reflects the glucose metabolic activity of the tissues and has an important role in the diagnosis and monitoring of malignant tumors (1). The PET has a high applicability for patients with breast tumors. Accordingly, the FDG uptake is reported to correlate with the malignancy grade and proliferative capacity of the tumors, as well as prognosis of the patients (2, 3). 

There is a body of evidence suggesting that the degree of FDG accumulation on PET/computed tomography (CT) is an independent predictor of disease-free survival (4, 5). However, the detectability of conventional PET/CT is limited to the lesions of more than 10 mm in size due to its low spatial resolution and partial volume effect (6-8).

As a result, positron emission mammography (PEM) was developed to overcome the low spatial resolution of PET/CT (8-11). Previous studies have reported a sensitivity of 78.6-95% and specificity of 86-90.6% for this modality in the diagnosis of breast cancer (6, 8-10, 12, 13). In a meta-analysis of eight published articles investigating the diagnostic capability of PEM, this imaging test was reported to have both high sensitivity (85%; 95% CI: 83%-88%) and high specificity (79%; 95% CI: 74%-83%) for breast cancer. However, when the mentioned analysis was focused on the tumors of ≤ l0 mm, there was little evidence regarding the diagnostic ability of this modality (14). 

A couple of reports showed a superior sensitivity of PEM as compared to that of PET/CT for the diagnosis of breast tumors of ≤ 10 mm (12, 13). On the other hand, several studies have failed to demonstrate the significant superiority of PEM over PET/CT in this regard (6, 15). With this background in mind, the present study was conducted to investigate the sensitivity, specificity, positive predictive value (PPV), negative predictive value (NPV), and accuracy of ^18^F-FDG PET/CT alone, PEM alone, and combined PET/CT and PEM (PET/CT+PEM) in the diagnosis of the breast tumors of ≤ 20 mm in size. 

## Methods

***Patients***

The study population corresponded to a group of 50 consecutive patients with biopsy-proven breast cancer having tumors of ≤ 20 mm as determined by ultrasonography. The patients were referred from the Department of Breast Surgery, Tohoku Kosai Hospital, Sendai, Japan, to Sendai Medical Imaging Clinic for undergoing whole body PET/CT and PEM imaging between March 2016 and April 2017. 

The control group consisted of 50 women who visited the imaging clinic for receiving routine medical checkup between August 2011 and August 2015. The control subjects were selected from the first 50 consecutive subjects undergoing imaging. The subjects who were not detected with breast cancer both at the first and second visits, occurring with an interval of at least one year, were included in the study as the control group. 

Consequently, this retrospective study was conducted on a total of 100 females (i.e., 200 breasts). The exclusion criteria were: 1) pregnancy or lactation, 2) history of breast operation, chemotherapy, radiotherapy, hormonal therapy, or mammary implant, and 3) a blood glucose level of ≥ 150 mg/dL. 

***Positron emission mammography***

The PEM was performed by means of a large-field (200×150 mm) planar-type dedicated breast PET scanner (PEMGRAPH, Furukawa Scintitec Co. Ltd., Iwaki, Japan) (16, 17). This scanner has eight detector blocks in two opposing detector paddles that immobilize the breast tissue during the image acquisition. Each detector paddle is comprised of 5120 pixelated scintillator crystals made of Pr3+-doped transparent ceramic lutetium aluminum garnet (Pr: LuAG) combined with H8500 position sensitive photomultipliers (PSPMTs). 

In this scanner, the crystal size is 2.1×2.1×15.0 mm, and the field of view is 200×150 mm². The time window and energy window were set at 6.0 ns and 450-580 keV, respectively. To perform this test, the patient sits vertically while her breast is between the paddles. There is a mobile thin acryl-made panel to fix the breast tissue and minimize body movement. The panel can mobile from 50 mm to 250 mm. 

The scanning process took about 3-4 min based on the preliminary phantom studies revealing that the lesion detectability was equivalent among the data acquisition times of 3, 10, and 20 min. The images were reconstructed by means of the 3D-Maximum Likelihood Expectation Maximization algorithm using eight iterations and an antialiasing filter with dead time, random and decay correction, and no attenuation correction. 

The images were reconstructed with a matrix size of 180×180, a pixel spacing of 1.1×1.1 mm, and a slice thickness of 3 mm. In a previous study, the spatial resolution was 2.1 mm as full width at half maximum (FWHM) measured by using a ^22^Na point source in the air, which placed the mid plane parallel to the detector face (18).

***Scan acquisition***

Prior to the implementation of PET/CT and PEM, the patients were required to undergo at least mammography and ultrasonography. The nuclear medicine doctors were informed about the tumor size in the maximum dimension and location in the breast quadrant prior to the imaging. The patients who had fasted for at least 5 h prior to the examination were administrated ^18^F -FDG (~3.7 MBq/kg) by intravenous injection via the cubital vein. 

In the breast cancer patients, conventional PET/CT imaging was performed about 75 min after the FDG injection in a combined PET/CT scanner (Biograph16, Siemens, Erlangen, Germany) for 2 min/bed position. The images were reconstructed by means of an iterative three-dimensional (3D) ordered-subsets expectation maximization (OSEM) algorithm using two iterations, eight subsets, and a 5-mm Gaussian filter with segmented attenuation correction. The reconstruction was performed with a matrix size of 168×168, a pixel spacing of 4.3×4.3 mm, and a slice thickness of 3 mm. 

The effective spatial resolution of PET/CT was 6.6 mm FWHM in the clinical setting at the Sendai Medical Imaging Clinic (19). The PEM was performed after the execution of PET/CT, about 100 min after the FDG injection, using a large-field (200×150 mm) planar-type dedicated breast PET scanner (PEMGRAPH, Furukawa Scintitec Co. Ltd., Iwaki, Japan). 

The mediolateral view images of each breast were obtained for 3-4 min, with the subjects sitting vertically on a chair. The distance between the detectors varied from 100-200 mm according to the size of the breast and location of the tumor. In the control subjects, PET/CT images were acquired 60 min after the intravenous injection of the tracer, and the PEM images were acquired in the same position 85 min after the injection.

***Image analysis***

Two experienced nuclear medicine physicians (FY, JH) who were blinded to clinical information about the subjects assessed the PET/CT and PEM images for the presence or absence of focal FDG uptake. In order to eliminate the reading bias, the PET/CT and PEM images were interpreted in a random order with the examiners blinded to the findings of the other imaging examinations. 

On each PET/CT and PEM image, the lesions demonstrating distinctly greater FDG uptake relative to the surrounding normal breast tissue were defined as showing positive FDG uptake. No FDG accumulation, or equivocal accumulation relative to the surrounding tissue was defined as negative. All disagreements with the readers were resolved by consensus. 

For all cases, the final diagnoses were compared with the pathological diagnoses with biopsy specimen. The sensitivities, specificities, PPVs, NPVs, and accuracies of PET/CT, PEM, and PET/CT+PEM were examined using both patient- and breast-based analyses. Subsequently, the sensitivities of PET/CT and PEM were compared for tumors measuring ≤ 10 and > 10 mm in size. 

***Phantom study***

The partial volume effects in PET/CT and PEM were estimated by applying 3D Gaussian filters on several 3D sphere digital phantoms of different diameters (i.e., 3, 4, 5, 8, 12, 18, and 25 mm). The count contrast between the spheres and background was set at 4 to 1. In actual phantom experiments, the spheres were blurred by applying a Gaussian filter with FWHM of 6.6, 6.6, and 6.6 mm for PET and 2.1, 2.1, and 6.0 mm for PEM. Since our PEM had anisotropic resolution, different FWHMs were used for each direction. The maximum count of the seven spheres was used to estimate the partial volume effects.

***Statistical analysis***

Quantitative data, such as age, weight, blood sugar levels, and tumor size, were expressed as mean, standard deviation, and range. For visual analysis, differences between PET/CT+PEM and PET/CT and between PET/CT and PEM were assessed using the McNemar’s test. All statistical analyses were performed in JMP, version 13 (SAS Institute Inc., Cary, NC, USA). P-value less than 0.05 was considered statistically significant. 

## Results

Table 1 summarizes the clinical information of the patients and controls. The total number of tumors was 54. One patient was diagnosed with bilateral breast cancer, and two patients were detected with multiple malignant breast tumors in the same mammary gland (i.e., two tumors in one case, three tumors in the other). The pathological features and size of the tumors are presented in Table 2. Based on the Union for International Cancer Control, the breast tumors were classified by size into three groups of T1a (>1 to ≤5 mm), T1b (>5 to ≤10 mm), and T1c (>10 to ≤20 mm). 

In the patient-based analysis, the results of visual image interpretation revealed that PET/CT, PEM, and PET/CT+PEM had the overall sensitivities of 60% (30/50), 72% (36/50), and 76% (38/50), specificities of 100% (50/50), 98% (49/50), and 98% (49/50), PPVs of 100% (30/30), 97.3% (36/37), and 97.4% (38/39), NPVs of 71.4% (50/70), 77.8% (49/63), and 80.3% (49/61), and accuracies of 80% (80/100), 85% (85/100), and 87% (87/100), respectively. The PET/CT+PEM showed significantly higher sensitivity and accuracy for the diagnosis of breast cancer, compared to PET/CT (P=0.005 and P=0.02, respectively). However, no difference was observed between PEM and PET/CT in this regard (Table 3). 

In the breast-based analysis, the results of visual image interpretation revealed that PET/CT, PEM, and PET/CT+PEM had the overall sensitivities of 58.8% (30/51), 70.6% (36/51), and 74.5% (38/51), NPVs of 70% (49/70), 76.6% (49/64), and 79% (49/62), and accuracies of 79% (79/100), 85% (85/100), and 87% (87/100), respectively. In addition, all modalities had the specificities and PPVs of 100%. The PET/CT+PEM showed significantly higher sensitivity and accuracy for the diagnosis of breast cancer in comparison to PET/CT (both P=0.005). Nonetheless, no difference was detected between PEM and PET/CT in this respect (Table 4).

**Figure 1:**
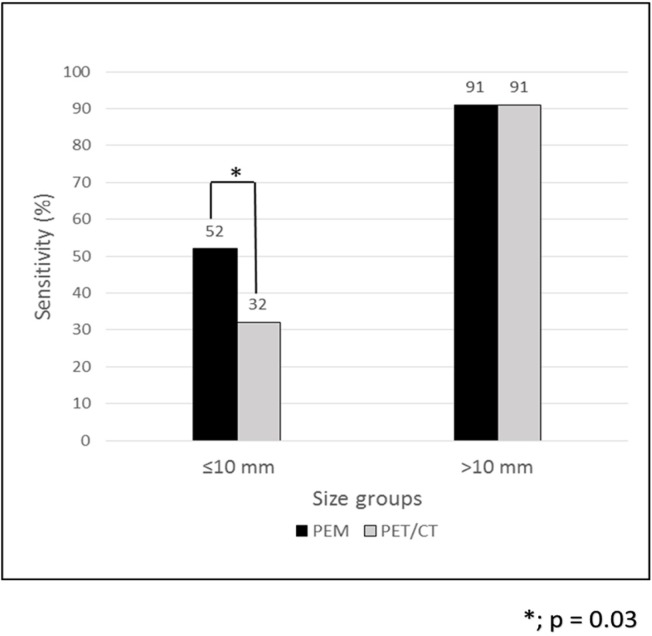
Tumor-based analysis to compare the sensitivities of PET/CT and PEM for the diagnosis of breast cancer. PEM allowed for the detection of a larger number of tumors as compared to PET/CT in the tumors of ≤ 10 mm in size. However, no significant difference was observed between these two modalities regarding the percentage of the tumors detected in the group of tumors of > 10 mm in size

The sensitivities of PET/CT and PEM were investigated by tumor size. The sensitivities of PEM for T1a, T1b, and T1c tumors were 50% (3/6), 52% (13/25), and 91% (21/23), respectively. However, PET/CT had the sensitivities of 0% (0/6), 40% (10/25), and 91% (21/23) for these tumors, respectively. When the tumors were classified into two groups according to the their size (i.e., ≤10 mm and >10 mm), the sensitivity of PEM was significantly higher than that of PET/CT in the ≤ 10 mm group (52% [16/31] vs. 32% [10/31]; P=0.03). However, no significant difference was found between the two modalities in the > 10 mm group (91% [21/23] for both modalities; P=1.0) (Figure 1). 

**Figure 2:**
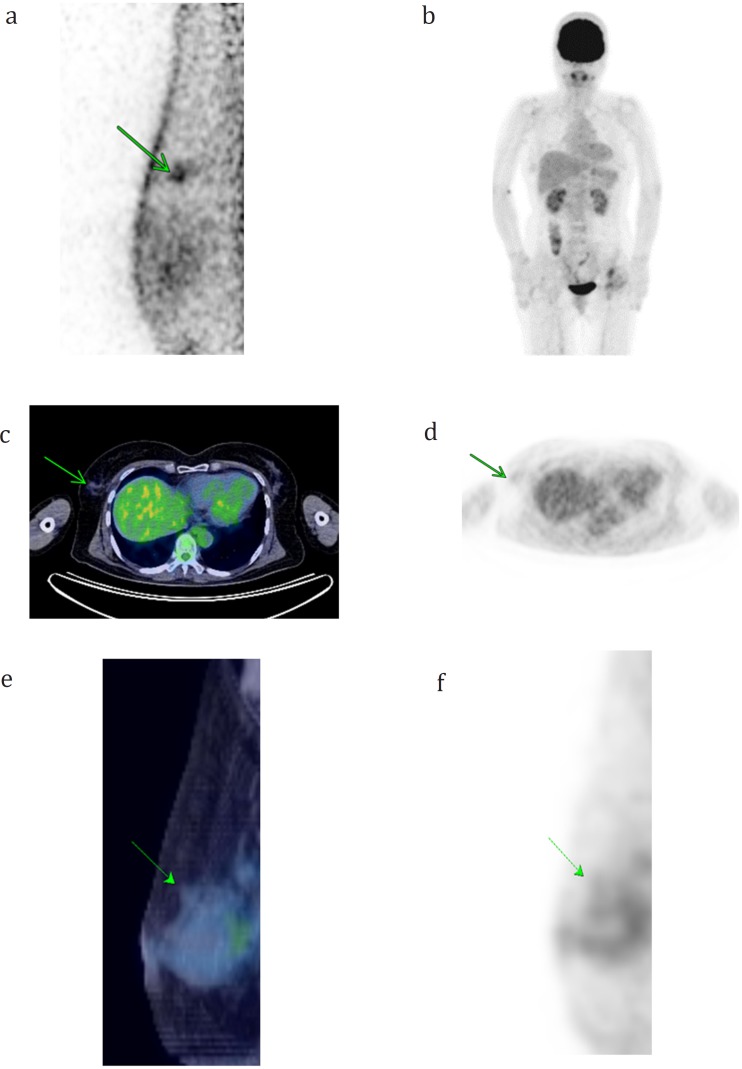
A 63-year-old female with an invasive ductal carcinoma measuring 4 mm in size (T1a) in the right breast. A medio-lateral PEM image (a) shows the focus of accumulation, whereas none of the MIP images of PET (b), axial images of fusion PET/CT (c) and PET (d) or sagittal images of fusion PET/CT (e) and PET (f) identified any focal FDG uptake in the right breast

Eight tumors could be only visualized by PEM (mean size: 7.8±3.7 mm, range: 4-15 mm). Figure 2 displays a representative case of a tumor that could be only visualized by PEM. In addition, there were two tumors that could be only visualized by PET/CT. They located just above the pectoral muscles, and it was speculated that they were outside of the field-of-views. 

**Figure 3:**
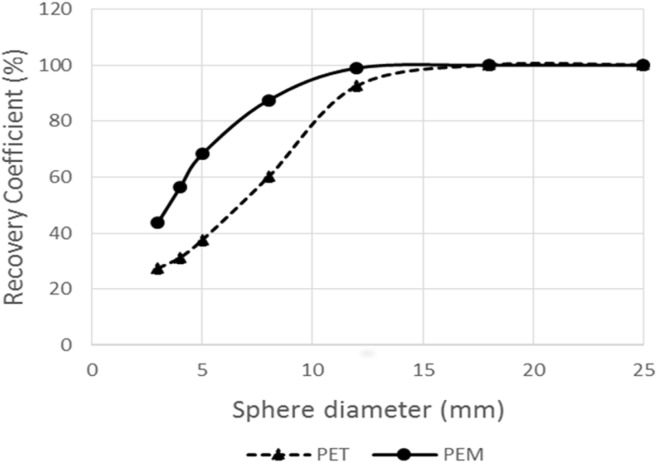
Correlation curve between the partial volume effects against size in PEM and PET in the simulating phantom study. PEM was associated with a smaller partial volume count loss than PET in lesions measuring ≤ 12 mm in size

One case in the controls showed focal accumulation with PEM revealing false positive by imaging follow-up for three years. Furthermore, in our simulation study, PEM showed higher percentage of recovery coefficients in lesions of ≤ 12 mm in diameter, compared to PET (Figure 3).

## Discussion

***Detectability of small breast cancers by PEM***

Most of the previous studies included patients with large breast tumors of 20 mm or more in size (8, 10, 20, 21). The sensitivity and specificity of PEM in these studies were reported as 90-96% and 84-91%, respectively. In the current study, we focused on T1-sized breast cancer with less than 20 mm in size. The PEM sensitivity obtained in the present study (70.6%) was much lower than those of the previous studies. 

The PEM showed a sensitivity of 52% (16/31) for T1a and T1b tumors of < 10 mm in size. In a recent study investigating PEM using a hanging-type dedicated breast PET scanner, the PEM allowed for the detection of 57% (13/23) of the tumors of ≤ 10 mm in size (15). In line with the other studies, the results of the present study indicated that PEM had a limited capability for detecting small breast cancer, especially when tumor size is less than 10 mm.

**Table 1:** Baseline characteristics of the participants

	**Breast cancer (n=50)**	**Control (n=50)**
**Age (year)**	57±11.8 (28-81)	57±9.2 (39-78)
**Weight (kg)**	56±7.8 (36.4-74.8)	54±7.7 (41.1-72.1)
**Blood Sugar (mg/dL)**	104±12.1 (78-141)	99±11.5 (80-126)
**Premenopausal**	n=16	n=11
**Postmenopausal**	n=33	n=38

**Table 2:** Characteristics of 54 tumors in 50 patients

**Pathologic diagnosis**	Invasive ductal carcinoma Invasive lobular carcinoma Mucinous carcinoma Apocrine carcinoma	47 3 3 1
**Size** *	T1a (>1 to ≤5 mm) T1b (>5 to ≤10 mm) T1c (>10 to ≤20 mm)	6 25 23

* UICC classification (Brierley JD, et al, eds: TNM classification of malignant tumors. 8th ed. UICC, 2016.)

**Table 3:** Patient-based analysis of the PET/CT, PEM, and PET/CT+PEM images by visual inspection

	**PET/CT**	**PEM**	**P-value** * **(vs. PET/CT)**	**PET/CT+PEM**	**P-value** * **(vs. PET/CT)**
**Sensitivity **	60% (30/50)	72% (36/50)	0.06	76% (38/50)	0.005
**Specificity**	100% (50/50)	98% (49/50)	0.3	98% (49/50)	0.3
**Positive predictive value**	100% (30/30)	97.3% (36/37)	－	97.4% (38/39)	－
**Negative predictive value**	71.4% (50/70)	77.8% (49/63)	－	80.3% (49/61)	－
**Accuracy**	80% (80/100)	85% (85/100)	0.1	87% (87/100)	0.02

* McNamar’s test

**Table 4:** Breast-based analysis of the PET/CT, PEM and PET/CT+PEM images by visual inspection

	**PET/CT**	**PEM**	**P-value** * **(vs. PET/CT)**	**PET/CT+PEM**	**P-value** * **(vs. PET/CT)**
**Sensitivity **	58.8% (30/51)	70.6% (36/51)	0.06	74.5% (38/51)	0.005
**Specificity**	100% (49/49)	100% (49/49)	-	100% (49/49)	-
**Positive predictive value**	100% (30/30)	100% (36/36)	-	100% (38/38)	-
**Negative predictive value**	70% (49/70)	76.6% (49/64)	--	79% (49/62)	-
**Accuracy**	79% (79/100)	85% (85/100)	0.06	87% (87/100)	0.005

* McNemar’s test

***Comparison of PEM with PET/CT***

In the present study, we compared the diagnostic performance of PEM with that of the PET/CT. As the results indicated, the sensitivity of PET/CT (60%) was much lower than that of the PEM (72%). This is due to the low sensitivity of the PET/CT in small tumors with less than 10 mm in size. When the tumor size was less than 5 mm, no breast cancer was detected by the PET/CT. Only 40% of the tumors were detected by PET/CT when the tumor size ranged within 5-10 mm. According to the results, the sensitivity of PEM (52%) was limited; however, it was superior to that of the PET/CT (10/31, 32%) for the tumors of less than 10 mm in size.

In the current study, two patients showed false negative results in the PEM. The tumors in these cases were located close to the chest wall outside the field-of-view of the PEM scanner. These tumors were detected only by PET/CT. The digital phantom analysis in the present study revealed the limited but better capability of PEM than PET/CT in detecting small-sized breast cancers. As shown in Figure 3, the underestimation of radioactivity concentration due to partial volume effect became large for the sphere phantoms of < 12 mm in diameter.

***Comparison of combined PET/CT and PEM with PET/CT alone***

In this study, combined PET/CT and PEM studies (PET/CT+PEM) showed significantly higher sensitivity and accuracy for the diagnosis of breast cancer, compared to PET/CT alone. This is probably due to the fact that PEM has a high sensitivity to small tumors of less than 10 mm in size, whereas the PET/CT has a large field-of-view that is not covered by the current PEM scanner. In the clinical setting, PEM could be applied in combination with PET/CT after a single injection of FDG.

***Research Limitations***

This study had several limitations. First, this is a retrospective study with relatively small sample size. Second, there is a bias of patient selection. Our study did not contain either ductal carcinoma in situ (DCIS) or any benign lesions with FDG-avidity, such as fibroadenoma or fibrocystic change (7, 8). Since the patients were referred to our clinic for staging of breast cancer to detect metastatic lesions, PEM study was performed in addition to whole body PET examination. 

Patients with DCIS lesion would not be referred to the whole body FDG PET/CT study for staging. The detectability of DCIS lesions of breast cancer by means of the PEM remains unknown. In addition, the absence of benign lesion with FDG-avidity would result in the overestimation of the specificity in this study. Finally, there was a difference in scan timing between the PET/CT and PEM; in this regard, PET/CT study was followed by PEM study after 20-30 min. 

According to the previous reports, the detectability of breast cancer may be improved in the delayed imaging (22). Accordingly, in a study, when dual time point imaging was employed, the sensitivity and accuracy of FDG PET/CT for breast cancer was reported to be much better in delayed imaging than in initial imaging, with an increase in the sensitivity of the tumors measuring ≤ 10 mm in size (23). Therefore, the implementation of PEM after PET/CT may cause benefit to the PEM in terms of detectability. 

## Conclusion

The present study demonstrated that PEM had limited capability in detecting breast cancer, especially when tumor size was less than 10 mm in size. Furthermore, combined PET/CT and PEM showed better diagnostic capability, compared with PET/CT alone. Regarding this, further studies are needed to prove the merits of PEM, especially for DCIS type of breast cancers.
